# Pro-resolving lipid mediator Resolvin D1 serves as a marker of lung disease in cystic fibrosis

**DOI:** 10.1371/journal.pone.0171249

**Published:** 2017-02-03

**Authors:** Olaf Eickmeier, Daniela Fussbroich, Klaus Mueller, Friederike Serve, Christina Smaczny, Stefan Zielen, Ralf Schubert

**Affiliations:** 1 Department for Children and Adolescents, Division of Allergology, Pulmonology, and Cystic Fibrosis, Goethe-University, Frankfurt, Germany; 2 Department of Food Technology, University of Applied Sciences, Fulda, Germany; 3 Department of Internal Medicine III, Goethe-University, Frankfurt, Germany; National Jewish Health, UNITED STATES

## Abstract

**Background:**

Cystic fibrosis (CF) is an autosomal recessive genetic disorder that affects multiple organs, including the lungs, pancreas, liver and intestine. Mutations in the cystic fibrosis transmembrane conductance regulator (CFTR) locus lead to defective proteins and reduced Cl^-^ secretion and Na^+^ hyperabsorption in the affected organs. In addition, patients suffering from CF display chronic inflammation that contributes to the pathogenesis of CF. Recent work suggests that CF patients have a reduced capacity to biosynthesize specialized pro-resolving lipid mediators (SPMs), which contributes to the development and duration of the unwanted inflammation. Alterations in the metabolism of arachidonic acid (AA) and docosahexaenoic acid (DHA) to specialized pro-resolving mediators (SPMs), like lipoxins (LXs), maresins (MaRs), protectins (PDs) and resolvins (Rvs), may play a major role on clinical impact of airway inflammation in CF.

**Methods:**

In this study, our aims were to detect and quantitate Resolvin D1 (RvD1) in sputum and plasma from patients with CF and compare levels of RvD1 with biomarkers of inflammation and lung function. We studied 27 CF patients aged 6 to 55 years (median 16 years) in a prospective approach.

**Results:**

DHA can be found in the plasma of our CF patients in the milligram range and is decreased in comparison to a healthy control group. The DHA-derived pro-resolving mediator Resolvin D1 (RvD1) was also present in the plasma (286.4 ± 50 pg/ mL, mean ± SEM) and sputum (30.0 ± 2.6 pg/ mL, mean ± SEM) samples from our patients with CF and showed a positive correlation with sputum inflammatory markers. The plasma concentrations of RvD1 were ten times higher than sputum concentrations. Interestingly, sputum RvD1/ IL-8 levels showed a positive correlation with FEV_1_ (r_s_ = 0.3962, p< 0.05).

**Conclusions:**

SPMs, like RvD1, are well known to down-regulate inflammatory pathways. Our study shows that the bioactive lipid mediator RvD1, derived from DHA, was present in sputum and plasma of CF patients and may serve as a representative peripheral biomarker of the lung resolution program for CF patients.

## Introduction

Cystic fibrosis (CF) is an autosomal recessive genetic disorder that affects multiple organs, including the lungs, pancreas, liver, and intestine. Cystic fibrosis is caused by the mutation of the gene coding for the Cystic Fibrosis Transmembrane Conductance Regulator (CFTR), a cyclic AMP-dependent Cl^-^ channel. The major clinical features of CF are chronic pulmonary disease, exocrine pancreatic insufficiency and male infertility [1, 2]. The lung disease is the main cause of morbidity and mortality in CF [3, 4]. The airway epithelium of patients with CF fails to transport Cl^-^, HCO_3_^-^ and water, resulting in a reduced airway surface liquid (ASL) height and impaired mucociliary clearance. The hyperabsorption of electrolytes observed in the CF bronchial epithelium might further aggravate the dehydration of the ASL. Mutations in CFTR gene cause defective Cl^-^ secretion and Na^+^ hyperabsorption by airway epithelia cells [5–7]. CFTR is also found on cells of the immune system, such as neutrophils [8], monocytes [9], and T cells [10], where loss of CFTR function leads to abnormal immune cellular function. Platelets from CF patients are affected by the molecular defect of CFTR and may play a role in the failure of resolution of inflammation in CF [11]. Mutations in CFTR lead to abnormally thick mucus [12] and aberrant immune responses [13, 14]. The net result is a propensity in CF patients for recurrent infections and over-exuberant, yet ineffective leukocyte recruitment, phagocytosis, killing, and clearance of pathogens. In comparison to other chronic inflammatory lung diseases such as chronic obstructive pulmonary disease (COPD) and asthma, CF is the leading disease in terms of uncontrolled inflammation [15–18]. So far, with the exception of high-dose ibuprofen, there is no anti- inflammatory treatment available for patients. It is well known for more than a decade that patients with cystic fibrosis have altered levels of plasma fatty acids. Previous studies demonstrated that arachidonic acid (AA) levels are increased and docosahexaenoic acid (DHA) levels are decreased in affected tissues from cystic fibrosis [19]. Recently, it has become evident that resolution of inflammation is a biochemically active process regulated in part by endogenous polyunsaturated fatty acid (PUFA)-derived mediators [20]. Novel families of mediators including lipoxins, resolvins, protectins and maresins represent a new genus of specialized pro- resolving mediators (SPMs) [21]. It has been demonstrated that an active pro-resolving lipid mediator derived from the Ω-6 fatty acid AA, namely Lipoxin A_4_ (LXA_4_), was decreased in the airways in patients with CF [6, 22, 23]. Lipoxins are bioactive lipids derived from Ω-6 PUFAs and play important roles in various biological functions [24]. To our best knowledge, there is no data about clinical status and the role of active lipid mediators derived from the Ω-3 PUFA DHA, namely RvD1 in cystic fibrosis lung disease. The endogenous RvD1 (7S, 8R,17S-trihydroxy-4Z,9E,11E,13Z,15E,19Z-docosahexaenoic acid) is produced at inflammatory sites from the interaction of lipoxygenase activities of several cell types including activated neutrophils, platelets and epithelial cells. RvD1 is one member of the newly identified lipid molecules promoting resolution of inflammatory processes by modulating neutrophilic inflammation, clearing apoptotic PMN and inhibiting the production of pro-inflammatory cytokines/ chemokines such as IL-8 [25].

RvD1 levels in CF airways could be a contributing factor in chronic airway inflammation which characterises these patients. There is no data available about the role of RvD1 in the airways of CF patients. In this study, we have investigated the levels of PUFAs, and in particular the pro-resolving lipid mediator, RvD1, in plasma and sputum of CF patients in comparison to inflammatory biomarkers. Furthermore, we investigated RvD1 levels in relation to clinical consequences in terms of lung function and *Pseudomonas* infection status.

## Materials and methods

### 2.1 Subjects and selection

Written informed consent was obtained from all participants. The study was approved by the local institutional ethics committee of the University Hospital Frankfurt. Patients were recruited from the Christiane Herzog CF- Center of the University Hospital Frankfurt, Germany. The population of this study consisted of 27 clinically stable patients with cystic fibrosis (8 were *P*. *aeruginosa*-infected) and 14 non-smoking healthy control subjects. Clinical stability was defined as absence of acute exacerbation of disease six weeks prior to inclusion. Acute exacerbation was defined by two of the following symptoms: Fever >38.0°C, increase of sputum, significant increase of C—reactive protein (CrP) and significant weight loss. Exclusion criteria were absence of current use of systemic antibiotic and steroid treatment, clinically relevant renal, cardiac, or hepatic (ALT/AST > 3 times of the upper normal limit, portal hypertension) dysfunction, chronic gastrointestinal disease not related to CF and pregnancy.

### 2.2 Sample collection and processing

Subjects first inhaled 200 μg Salbutamol and subsequent nebulised hypertonic saline at concentrations of 3%, 4% and 5% for every 7 minutes. This bronchial stimulus caused expectoration of sputum. Before inhalation of hypertonic saline, the mouth was cleaned by flushing with water. Sputum was processed within 2 hours of collection [26]. The selected sputum plugs were as far as possible without saliva, processed into a weighed Eppendorf tube and processed with 2× weight/volume of 0.1% Dithiothreitol (DTT). Afterwards 4× weight/volume of PBS was added. Samples were filtered through 48 μm mesh and centrifuged for 10 minutes at 790× g to remove the cells. Supernatants were stored at -80C° until further analysis.

### 2.3 Cytometric bead array

Concentrations of different cytokines/chemokines were determined in sputum samples using the BD^™^ CBA Flex Set System for the measurement of IL-1β and IL-8 (BD Bioscience-PharMingen, San Diego, CA, USA) levels. Each BD^™^ CBA Flex Set contained one bead population with distinct fluorescence intensity, as well as the appropriate phycoerythrin (PE) detection reagent and standard. The tests were performed according to the manufacturer’s advice, and samples were run in duplicate [27, 28]. For analyses of the cytokines/ chemokines, we added the same concentration of DTT (0.025%) as in the sputum supernatant to the standard curve and enzyme immunoassay buffer.

### 2.4 Biochemical analysis

DHA and AA measurement in blood plasma were performed by fatty acid methyl esters (FAME) analysis. Total lipids of plasma and cruor were extracted according to Bligh and Dyer [29]. For derivatization, plasma was dissolved in 1000 μL petroleum ether followed by addition of 50 μL sodium methoxide-solution in accordance with the method of Kohn [30]. The organic solvent was completely evaporated, overlaid with nitrogen and stored at -80°C until measurement. The samples were than analysed by capillary gas chromatography (CGC).

RvD1 concentrations were measured by ELISA technique using Greiner microtiter plates (Greiner Bio-One, Frickenhausen, Germany) according to the manufacturer´s instructions. Briefly, RvD1 was extracted from plasma and sputum via Sep-Pak C18 light cartridges (Waters, Eschborn, Germany). After equilibrating columns with methanol and washing with deionized water, samples diluted in 1M acetate buffer were applied to the column. Elution of RvD1 with ethyl acetate was conducted after second washing with deionized water. Solvent was evaporated under a gentle stream of nitrogen and the residue was immediately dissolved in sample buffer and subjected to ELISA.

### 2.5 Pulmonary function test

Pulmonary function tests were performed according to American Thoracic Society/ European Respiratory Society guidelines for performance and acceptance prior to sputum induction [31].

### 2.6 Statistical analysis

Data were analysed using the statistical program GraphPad Prism (GraphPad Software Inc., La Jolla, CA, USA) and Microsoft Excel (Microsoft Corporation, Redmond, WA, USA). Differences between the groups were performed by the nonparametric Kruskal-Wallis test or a Mann- Whitney test depending on Gaussian distribution and the homogeneity of the variances. Correlations between biomarkers and clinical parameters were verified by using Spearman correlation coefficient (r_s_). A probability of *p* < 0.05 was regarded as significant. In figures data are shown as individual values and mean ± SEM.

## Results

### Pulmonary function is associated with sputum neutrophil count

**Fig 1:**
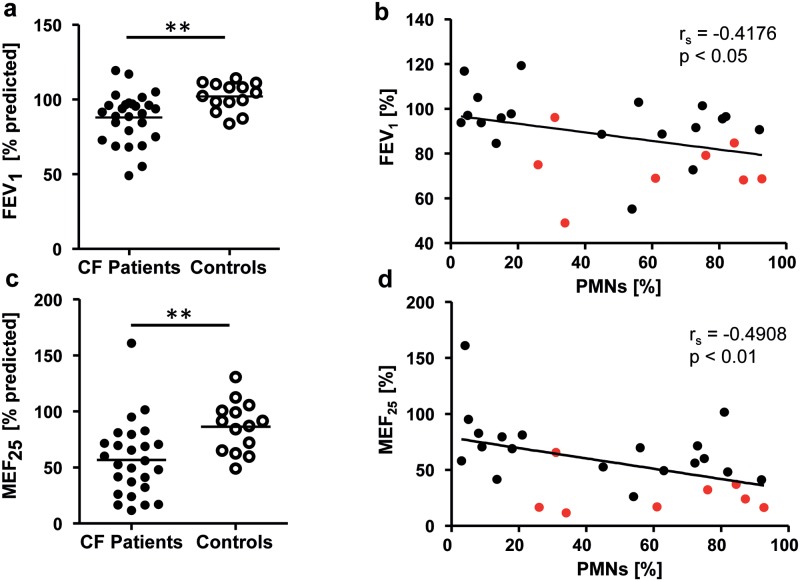
Baseline pulmonary function data of CF patients and healthy controls and correlation of pulmonary function in CF to sputum neutrophils. Comparison of mean (a) FEV_1_ [%] and (c) MEF 25 [%] in CF patients and healthy controls. Correlation of (b) FEV_1_ [%] and (d) MEF25 [%] in CF patients with percentage of sputum neutrophils. Red dots represent *Ps*. *a*. positive patients. Values are represented as Mean ± SEM. The Spearman correlation coefficient (r_s_) and the associated p-value is given for each analysis. ** p< 0.01.

The demographic and baseline clinical characteristics of the subjects with cystic fibrosis and the healthy control subjects are shown in Table 1. The distribution of genotypes in our study cohort closely reflected the genotype distribution in our CF clinic population. The majority of our patients were pancreatic insufficient (92.6%). The minority of our patients (29.6%) were *Pseudomonas aeruginosa* positive. Even though the FEV_1_ (% predicted) of our CF patients ranged from 49 to 119% (Fig 1a), most of our subjects had normal or mildly abnormal lung function (Fig 1a) with a broad distribution of the flows in the small airways represented by MEF_25_ (%predicted) ranging from 11.6 to 161%predicted (Fig 1c). Furthermore, we were able to demonstrate a negative correlation between pulmonary function data and sputum neutrophils (Fig 1b and 1d). This is shown most prominently by decreased air flow in the small airways (MEF_25_) in correlation with sputum PMNs (p < 0.01).

**Table 1:** Patient characteristics.

	Healthy Controls	CF
Subjects	14	27
Sex [m/f]	8/ 6	14/ 13
Age	18.2 ± 4.4	17.5 ± 11.5
BMI [kg/ m^2^]	22.1 ± 3.5	18.6 ± 3.4
ΔF508 homozygous	-	13
Pancreatic insufficient	-	25
Ps. aeruginosa positive	-	8
FEV_1_ [%pred.]	102.1 ± 9.4	88.1 ± 16.7

### Plasma fatty acid level of docosahexaenoic acid (DHA) is decreased in CF

**Fig 2:**
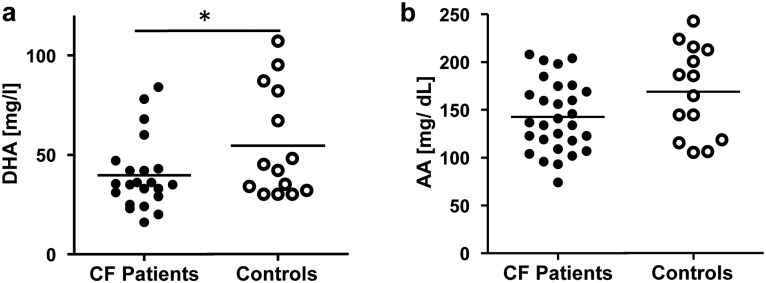
Levels of PUFAs in plasma from CF patients and healthy controls. Comparison of mean (a) DHA and (b) AA in plasma from CF patients and healthy controls. Values are represented as Mean ± SEM. * p< 0.05.

To investigate whether airway inflammation may also correlate with plasma fatty acid levels we determined fatty acid profiles in plasma. Indeed, DHA levels in plasma were decreased in our CF patients (37.2 ± 3.0 mg/dL, mean ± SEM) in comparison to the healthy control group (54.6 ± 7.3 mg/ dL, mean ± SEM, p< 0.05) (Fig 2a). No significant differences were measured for AA in plasma between both groups (Fig 2b).

### RvD1 is present in plasma and sputum of CF patients and levels of sputum RvD1 correlate with plasma RvD1 levels

**Fig 3:**
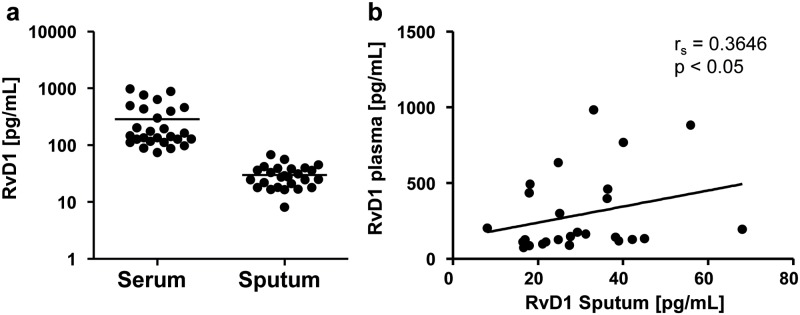
Levels of RvD1 in plasma and sputum from CF patients. Levels of (a) plasma and sputum RvD1 from CF patients and (b) correlation of plasma and sputum RvD1 levels. Values are represented as Mean ± SEM. The Spearman correlation coefficient (r_s_) and the associated p- value is given for each analysis.

To identify disturbances of pro-resolving mechanisms in CF resulting from differences in fatty acid levels, an active lipid mediator of DHA, namely RvD1, was measured in plasma and sputum of our CF patients. RvD1 was present in the picogram range in plasma (286.4 ± 50 pg/ mL, mean ± SEM) and sputum (30.0 ± 2.6 pg/ mL, mean ± SEM) (Fig 3a) of our CF patients and concentrations in plasma showed a positive correlation with concentrations in sputum (r_s_ = 0.3646, p < 0.05) (Fig 3b).

### RvD1 in CF sputum correlates with sputum and serum inflammatory biomarkers

**Fig 4:**
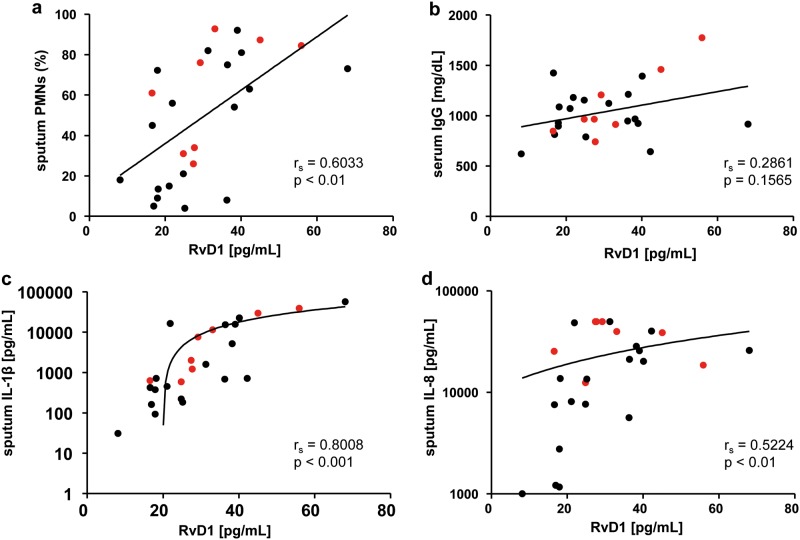
Correlation of pro-inflammatory sputum and serum biomarkers with sputum RvD1 levels. Correlation of (a) sputum PMNs, (b) serum IgG, (c) sputum IL-1β and (d) sputum IL-8 with sputum RvD1 levels from CF patients. Red dots represent *Ps*. *a*. positive patients. The Spearman correlation coefficient (r_s_) and the associated p- value is given for each analysis.

RvD1 is an anti-inflammatory and pro-resolving mediator, so correlation of RvD1 in sputum was performed with selected biomarkers of inflammation in sputum (Fig 4a, 4c and 4d) and serum (Fig 4b). Sputum RvD1 levels showed a significant positive correlation with sputum PMNs (r_s_ = 0.6033, p< 0.01; Fig 4a), sputum IL-1β levels (r_s_ = 0.8008, p< 0.001; Fig 4c) and sputum IL-8 levels (r_s_ = 0.5224, p< 0.01; Fig 4d). In addition, sputum Resolvin D1 showed a tangential positive correlation with serum IgG levels (Fig 4b).

### Increased RvD1/ IL-8 ratio in patients with CF is associated with a better pulmonary function

**Fig 5:**
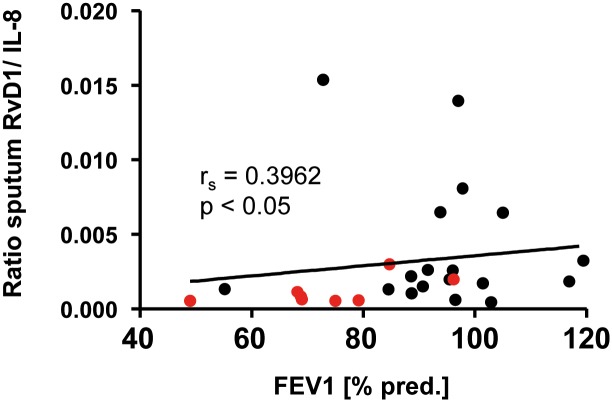
Correlation of sputum RvD1/ IL-8 ratio with FEV_1_. Correlation of sputum RvD1/ IL-8 ratio with FEV_1_ [%]. Red dots represent *Ps*. *a*. positive patients. The Spearman correlation coefficient (r_s_) and the associated p- value is given for each analysis.

With both pro- inflammatory marker IL-8 and pro-resolution marker RvD1 present in CF sputum, we determined the impact of RvD1/ IL-8 ratio on lung function. There was a significant positive correlation of the RvD1/ IL-8 ratio with FEV_1_ (r_s_ = 0.3962, p< 0.05) (Fig 5).

## Discussion

Progressive pulmonary disease is the major cause of morbidity and mortality in CF patients. CF lungs are challenged by an immense burden with pathogenic bacteria resulting in chronic lung infiltration with neutrophils and release of neutrophil elastase and other damaging products. This contributes negatively to bronchiectasis and pulmonary fibrosis [13, 32–34]. Furthermore, additional abnormalities in innate immune responses have been identified that lead to chronic inflammation and contribute to the pathogenesis of CF. These abnormalities lead to a decrease in anti-inflammatory lipid mediator production [6, 35] and an increase in pro-inflammatory chemokines, such as IL-8 [36–38]. Anti-inflammatory and pro-resolution treatment strategies are a new way of combatting chronic inflammation in CF. The use of SPMs as part of humanized nano-pro-resolving medicines is an exciting concept for this purpose. Hence, it is of critical importance to define the SPM profile in CF patients in order to address specific unmet needs by pharmacological intervention [21]. In previous studies, we have analysed specific pro-inflammatory markers in chronic and severe inflammatory lung diseases in order to detect pathognomonic patterns for the underlying disease [18, 38–45]. The knowledge that chronic inflammation is harmful to the CF lung has resulted in strategies to reduce lung infection [46] and the damage associated with chronic inflammation [47].

Patients with cystic fibrosis (CF] show decreased serum and sputum levels of linoleic acid (LA) and docosahexaenoic acid (DHA). The potential benefits of dietary addition of polyunsaturated fatty acids (PUFAs) in CF are still uncertain [48]. Supplementation of high doses of omega-3 fatty acids was effective in terms of correction of the lipid imbalance, but data on improvement in lung function and other clinical aspects are not conclusive [49]. Further investigation is needed to determine if altered metabolism of arachidonic acid and DHA to specialized pro-resolving mediators, like lipoxins (LX), maresins (MaR), protectins (PD) and resolvins (Rv), may impact the efficacy of supplementation therapy. Of interest, and in contrast to administration of PUFAs, like DHA and eicosapentaenoic acid (EPA), in the range of milligram to gram doses in past and ongoing clinical trials, the pro-resolving mediators (Rvs) are bioactive in the nanogram to picogram range [21]. In this study, we have been able to confirm decreased DHA levels in plasma of our CF patients in comparison to healthy controls. Evidence suggests that reduced DHA plasma levels could be due to suboptimal intestinal absorption of fatty acids in CF patients [50, 51]. Freedman et al. showed that DHA therapy leads to fewer neutrophils in the lungs of CF knock-out mice [50]. We employed a standardized and validated sputum-induction protocol to noninvasively study the local inflammatory responses [26]. We were able to detect RvD1 as a representative of DHA derived SPMs in plasma as well as the sputum of our CF patients.

Markers of inflammatory activity are important for the assessment and management of chronic respiratory diseases like CF and COPD [52, 53]. Numerous studies have analysed inflammatory cytokines in sputum, in relation to clinical status [54–56]. Until today, there is no study which has investigated the relation of inflammatory status, and lung function to RvD1 in CF patients. We demonstrate a positive correlation between sputum RvD1/ IL-8 levels and lung function. We have also shown that there is an abnormally low ratio of this pro-resolution mediator in comparison to pro-inflammatory biomarkers that correlates to decreased lung function. The ratio of Resolvin D1 to pro-inflammatory chemokine IL-8 is low in sputum of CF patients, even in the absence of an acute infection or exacerbation, strongly suggesting that there is an important pathophysiological effect for Resolvin anti-inflammatory and pro- resolution activity in the CF lung. SPMs like Resolvins can enhance the release of anti- microbial peptides. Of note, 30% of our CF group consisted of *Pseudomonas aeruginosa-* infected patients. CF patients who have higher levels of SPMs, like Resolvin D1 in the lungs have more preservation of FEV_1_ and fewer *Pseudomonas aeruginosa* infections.

In conclusion, our study shows that the bioactive lipid mediator RvD1, derived from DHA, was present in sputum and serum of CF patients and may serve as a representative peripheral biomarker of the lung resolution program for CF patients. This is the first study confirming a correlation between RvD1 levels with biomarkers, such as IL-1β or IL-8, in induced sputum in CF patients. Moreover, this study strongly indicated a positive correlation of sputum RvD1/ IL-8 levels with lung function data suggesting an important role for Resolvin-mediated anti-inflammatory and pro- resolution activity in the CF lung.
